# Beat-wise segmentation of electrocardiogram using adaptive windowing and deep neural network

**DOI:** 10.1038/s41598-023-37773-y

**Published:** 2023-07-07

**Authors:** S. M. Isuru Niroshana, Satoshi Kuroda, Kazuyuki Tanaka, Wenxi Chen

**Affiliations:** 1 Biomedical Information Engineering Lab, The University of Aizu, Fukushima, 965-8580 Japan; 2 Information System Engineering Inc.(ISE), Tokyo, 169-0075 Japan

**Keywords:** Cardiology, Health care, Health occupations, Medical research, Biomedical engineering

## Abstract

Timely detection of anomalies and automatic interpretation of an electrocardiogram (ECG) play a crucial role in many healthcare applications, such as patient monitoring and post treatments. Beat-wise segmentation is one of the essential steps in ensuring the confidence and fidelity of many automatic ECG classification methods. In this sense, we present a reliable ECG beat segmentation technique using a CNN model with an adaptive windowing algorithm. The proposed adaptive windowing algorithm can recognise cardiac cycle events and perform segmentation, including regular and irregular beats from an ECG signal with satisfactorily accurate boundaries.The proposed algorithm was evaluated quantitatively and qualitatively based on the annotations provided with the datasets and beat-wise manual inspection. The algorithm performed satisfactorily well for the MIT-BIH dataset with a 99.08% accuracy and a 99.08% of F1-score in detecting heartbeats along with a 99.25% of accuracy in determining correct boundaries. The proposed method successfully detected heartbeats from the European S-T database with a 98.3% accuracy and 97.4% precision. The algorithm showed 99.4% of accuracy and precision for Fantasia database. In summary, the algorithm’s overall performance on these three datasets suggests a high possibility of applying this algorithm in various applications in ECG analysis, including clinical applications with greater confidence.

## Introduction

A typical electrocardiogram (ECG) depicts the heart’s electrical activity and is a well-established cardiology technique for analysing the heart’s medical state and diagnosing heart anomalies. Careful examination of an ECG by an expert cardiologist or a physician is one of the standard practices in routine clinical procedures as ECG is recognised as a primary vital signal that ties with the physiology of the human body. The ECG beats’ regularity is also used as a diagnostic tool in specific topics such as evaluating mental stress^1,2^. However, the traditional diagnosis is becoming inefficient because, large amounts of heterogeneous data generated with the rapid spread of heart-related disorders in modern society. ECG inspection is essential to detect severe cases and perform close inspections after treatments, due to high prevalence of heart related complications^3,4^.

Various techniques have been proposed and implemented to perform automatic computer-based ECG classification in the past decades. Many follow three phases to perform the detection; (i) pre-processing, (ii) heartbeat segmentation (iii) beat-wise classification^5–7^. Automatic detection and segmentation of the ECG beat with R-peak (the critical event when detecting a single beat) is one of the essential steps in many ECG-based algorithms, including cardiac diagnosing^8,9^, heart rate variability analysis, and ECG-based authentication^10,11^. The importance of heartbeat segmentation becomes more pronounced in ECG analysis, where the classification phase strictly relies on the separated heartbeat^5^. Misdetections occur in the segmentation phase can propagate the error to the subsequent stages causing malfunction in the classification algorithms. Generally, these algorithms are designed based on digital filters^12–16^, signal processing techniques^17^, linear prediction, wavelet transforms^18–23^, derivatives, mathematical morphology^24,25^, geometrical matching, neural networks and hybrid approaches^26,27^. This article proposes two ECG beat segmentation methods using a CNN model and an adaptive windowing technique which can potentially employed as a preprocessing tool in beat-wise ECG analysing algorithms.

**Table 1:** Symbols and definitions used in this article.

Symbol	Definition
Variables	
$f_{s}$	Sampling rate
*j*	*j* *th* heartbeat
*i*	*i* *th* alternative ECG segment index (heartbeat and $\lnot$heartbeat)
*t*, *m*, *n*	Discrete-time variables (Time sampling)
*u*(*t*)	Discrete-time ECG signal comprises a successive heartbeat triplet (discrete-time variable *t*)
$v_{i,j}(m)$	$i{th}$ Variation of ECG segment extracted from $j^{th}$ Main heartbeat in *u*(*t*)
$L_{i,j}$	Length of $v_{i,j}(m)$
$w_{i,j}(n)$	Aligned version of $v_{i,j}(m)$
$\omega _{j}$	Updated window length after *j* *th* detecting *j* *th* heartbeat
$s_{j}$	Window stepping length (step size)
$cp_{j}$	$j^{th}$ Critical point (horizontal index)
$\tilde{cp_{j}}$	Temporary critical point before confirming the $cp_{j}$ (horizontal index)
$c_{j}$	The length between $cp_{j}$ and $cp_{j-1}$
${\overline{{\textbf {C}}}_{j}}$	Mean critical point interval
*wst*	Window starting position
$\overline{cp}_{{min}_{j}}$	Estimated value of minimum $cp_j$ (detects unusual *CP*s located too close)
$\overline{cp}_{{max}_{j}}$	Estimated value of maximum $cp_j$ (detects unusual $c_{j}s$)
${b}_{j}^l$	Left margin of the *j* *th* segmented beat (method I, immediate detection)
${b}_{j}^r$	Right margin of the *j* *th* segmented beat (method I, immediate detection)
$\tilde{{b}_{j}^l}$	Left margin of the $({j-1}){th}$ segmented beat (method II detection)
$\tilde{{b}_{j}^r}$	Right margin of the $({j-1}){th}$ segmented beat (method II)
Constants	
$\eta _w$	Regulates the windowing length
$\eta _s$	Regulates the step size
$\eta _{of}$	Regulates the offset length from the cp
$\eta _{sof}$	Regulates the length of small steps
$\eta _{cmin}$	Regulates estimated expected value of minimum $cp_j$
$\eta _{cmax}$	Regulates estimated expected value of maximum $cp_j$
$\eta _\delta$	Regulates the length of segmented ECG (safety margin)
$\eta _{ar}$	Aligning ratio
$p_b$	Set probability for detecting heartbeat
*M*	Fixed length of the input
*K*	Number of heartbeats used for moving average
Others	
$s^\delta$	Use small step if *true*
$b^+$	*True* if heartbeat is detected
$\zeta _{ECG}$	Raw ECG signal $\zeta _{ECG} \ge \omega _0$
$\zeta _{temp}$	Part of raw ECG signal extracted $0 < \zeta _{temp} \le M$
$\Theta ^{cnn}$	CNN model
$f^a()$	Function which aligns an ECG segment
$f^p()$	Function calculates the probability of being a heartbeat
$f^w()$	Function calculates all the adaptive window parameters
*P*(*B*)	Probability of being a heartbeat event (B)
$\delta$	Offset values calculated when $P(b) \ge p_b$ but No heartbeat detection

## Methods

In this section, the methodology for training the CNN to distinguish ECG heartbeats and the concept of the adaptive windowing algorithm are presented. Table 1 shows the symbols and definitions used in this article.

### Implementation of the CNN

#### Dataset, pre-processing, and augmentation

**Figure 1:**
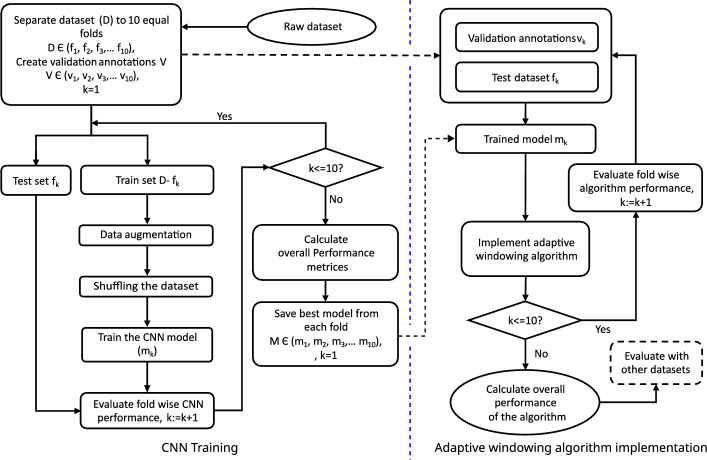
The method flow for the implementation of the proposed approach.

**Figure 2:**
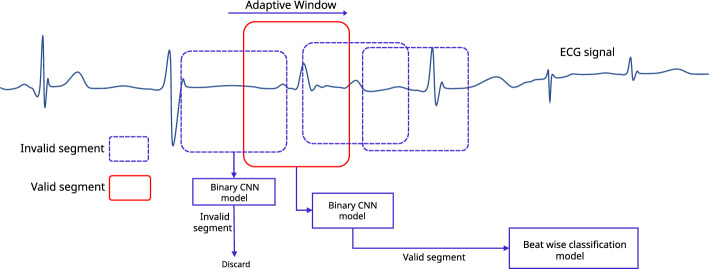
The overview of the proposed windowing approach.

The main steps followed for implementing the proposed adaptive windowing algorithm are shown in Fig. 1. Mainly it consists of two phases, (A). Implementation and validation of the CNN model based on k-fold cross-validation (B). Implementation and validation of the adaptive windowing (see Fig. 2). ECG data from the MIT-BIH arrhythmia database^28,29^ was employed to assess the proposed technique. The MIT-BIH arrhythmia database comprises diverse beat types derived from 48 recordings of 47 subjects, with each record containing a 30-min long ECG segment sampled at 360 Hz and band-pass filtered at 0.1–100 Hz. The dataset includes an annotation file for each record, specifying each R peak position and the heartbeat label. Each record contains an upper and lower lead signal acquired by placing the electrodes on the chest.

Adding random perturbation based on a meaningful augmentation strategy can increase the diversity of the dataset (variance). Data augmentation is employed not only as a potential method for improving performances in the speech and vision domains^30,31^ but also in ECG classification^32–34^.

**Figure 3:**
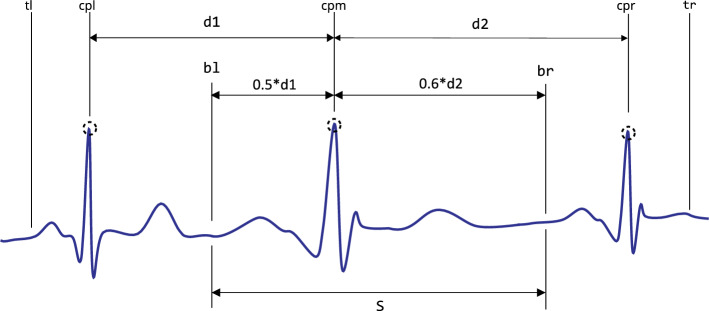
An ECG segment contains a triplet of critical points, *cpl* left critical point, *cpr* right critical point, *cpm* main critical point, *bl* left margin of the main beat, *br* right margin of the main beat, *tl* - left trimming point (*cpl* offset), *tr* right trimming point (*cpr* + offset), *s* length of the main ECG beat $(s = 0.5d1 + 0.6d2)$).

**Figure 4:**
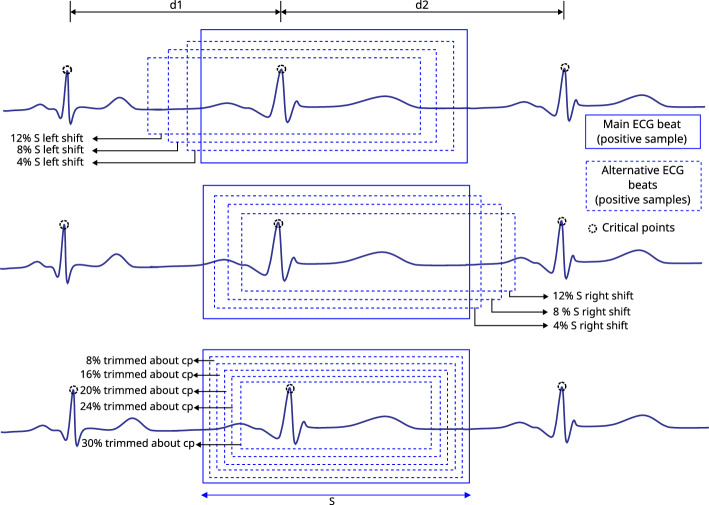
Creating 12 alternative positive ECG beat segments based on the main ECG beat in the middle of a triplet of ECG beats shown in Fig. 3, all the shifts and scale down are calculated reference to length **s**.

**Figure 5:**
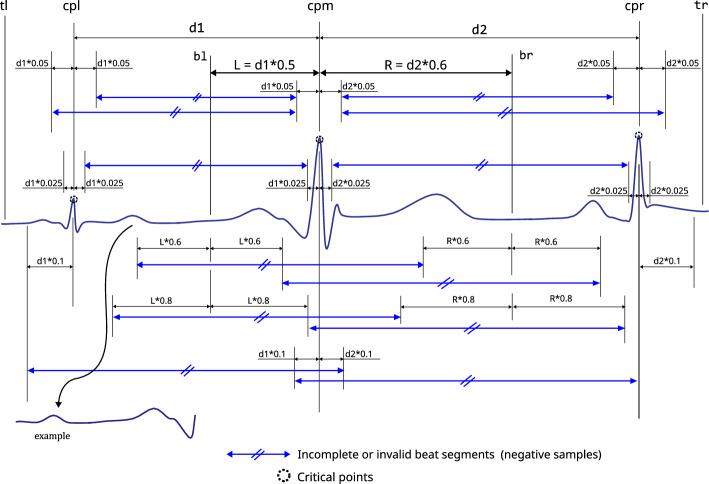
Creating 12 negative ECG beat segments based on adjacent critical points of a triplet of ECG beats shown in Fig. 3.

Before starting the CNN training, data augmentation was carried out as explained in Figs. 3, 4, and 5. The term **QRS-like** is used in the rest of the article to denote a typical or atypical cardiac cycle event, as QRS patterns may be distorted or not perfectly presented in abnormal ECG beats. Generally, CNN requires a specific fixed input size, considering the worst-case scenario, we set the input segment size as 512 samples ( $\approx 1.4\,s$ for signals sampled at 360 Hz).

To produce positive and negative samples, we exploited three critical points and other points as described in Fig. 3. Here, a positive sample means an ECG beat where elementary cardiac cycle attributes are seated vaguely in the centre [a segment $(\le 1.4\,s)$ comprised of rudimentary QRS characteristics where a normal or an abnormal cardiac beat event is presented]. Slightly shifted versions of the main **QRS-like** pattern were extracted to produce such alternative ECG-beat segments. The amounts of the shift to the right and left are 4, 8, and 12% of the relative length ***s***, respectively (where **s** is the length of the main **QRS-like** ECG segment as shown in Fig. 3). These shifted versions are adequately off-centred versions of the main **QRS-like** pattern and adequately off from being positioned too close to the rightmost or leftmost corners. Additionally, five end-trimmed versions around the main critical point were also created to ensure that the CNN can identify distorted versions of the main **QRS-like** pattern. 12 positive versions (11 augmented versions) of **QRS-like** patterns were created, including the main QRS morphology which is illustrated in Fig. 4. Having a robust CNN which can detect off-centred and shifted versions makes it easier to locate the ECG beat even if the window is not perfectly aligned with the main points.

A negative sample means an ECG segment where attributes of a complete cardiac cycle are not entirely presented or partially accommodated (segments $(\le 1.4\,s)$ which do not represent a normal or an abnormal cardiac beat event completely). Generating 12 negative samples using an ECG segment is shown in Fig. 5. Here, incomplete parts of **QRS-like** shapes, extremely left or right-shifted versions, and segments containing two critical points were extracted using consecutive critical points.

**Figure 6:**
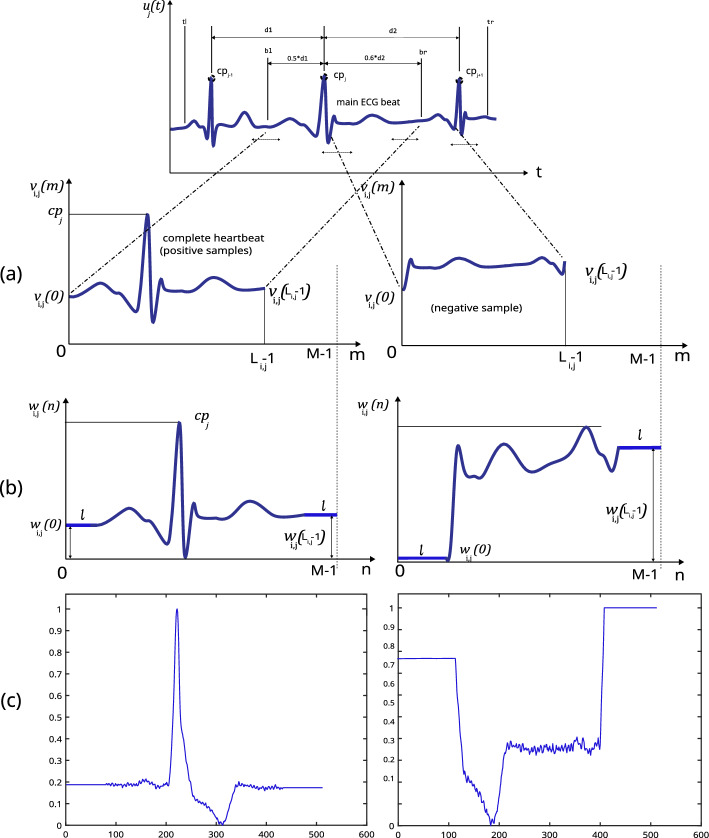
Homogenising the length of clipped ECG to a fixed length (512 samples), centralising the main ECG event and scale to [0,1] range. (**a**) Examples of the positive and negative sample (**b**) Examples of positive and negative samples after aligning (**c**). Example of the process applied to positive and negative samples from a record.

Any alternative positive or negative segment (separated according to Figs. 4 and 5) can be represented as signal $v_{i,j}(m)$ after separating ECG segment $u_{j}(t)$ (see Fig. 6a) with the main heartbeat $(H_j)$ using the adjacent critical points $(cp_{j-1},cp_{j},cp_{j+1})$. Here $v_{i,j}(m)$ is the $i^{th}$ alternative example from $j^{th}$ heartbeat with sample points designated as *m* as illustrated in Fig. 6a. The length of each segment is shown as $L_{i,j}$. As the $L_{i,j}$ can vary from segment to segment, all $v_{i,j}(m)$ are homogenised to have $M (=512)$ samples and aligned to form $w_{i,j}(n)$ as shown in Fig. 6b and in Eq. (1) to represent VBs and NVBs.

1 $$\begin{aligned} w_{i,j}(n)=\left\{ \begin{array}{l} v_{i,j}(0) \quad ; {if} \quad \ 0 \le n < \bigg \lceil \frac{M-L_{i,j}}{2}\bigg \rceil \\ v_{i,j}(L_{i,j}-1) \quad ;if \quad n \ge \bigg \lceil \frac{M-L_{i,j}}{2}\bigg \rceil + L_{i,j} \\ v_{i,j}\Bigl ( n-\bigg \lceil \frac{M-L_{i,j}}{2}\bigg \rceil \Bigr ) \quad ; {otherwise} \end{array} \right. \end{aligned}$$

where, $$x_{i,j}, \{ n, L, M\in \mathbb {Z}, n \ge 0,M \ge L_{i,j} >0 \}$$

2 $$\begin{aligned} x_{i,j}(n)= & {} \frac{w_{i,j}(n) - min(w_{i,j})}{max(w_{i,j})- min(w_{i,j})} \end{aligned}$$

3 $$\begin{aligned} x_{i,j}= & {} [x_{i,j}(0), x_{i,j}(1), x_{i,j}(2),\ldots , x_{i,j}(M-1)]^T \end{aligned}$$

After the centring alignment strategy was carried out, all $w_{i,j}$ were min-max normalised to the range [0, 1] to form vector $x_{i,j}$ with length $M(=512)$ as depicted in Eq. (2). As the original sampling rate $f_s$ is 360  Hz, the maximum length of the ECG segment is $\approx$1422 ms $(\frac{512}{360})$. The set of feature vectors in the dataset $\chi$ can be denoted as in Eq. (4)

4 $$\begin{aligned} \chi = \{x_{1,1}, x_{2,1}, x_{3,1},\dots ,x_{i,j},\dots ,x_{I,J}\} \end{aligned}$$

where, $(i,j \in \mathbb {Z}, I \ge i> 1, J > j \ge 0, I = 24)$, $J-$ total heartbeats, $j=0$ is undefined in the training phase, $I-$ total alternatives per beat.

In this study, an input segment $x_{i,j}$ (see Eq. (3)) from the training dataset $\chi$ can be denoted by $x_{i,j} \in \mathbb {R}_{M*1}$ with its label $y_{i,j} \in Y$, and $Y=\{VB, NVB\}$. Then, the proposed CNN model can be defined by a function $\hat{f}:x_{i,j}\rightarrow y_{i,j}$, which is later used to derive the function $f^{p}(\Theta ^{cnn}, \zeta _{temp})$.

#### CNN training and evaluation

The CNN model architecture comprises five convolutional layers followed by rectified linear unit (ReLU) activation and max pooling layers. Finally, a fully-connected layer is followed by a dropout layer and a SoftMax layer for binary classification.

The proposed CNN was implemented in MATLAB 2021a using record-wise 10-fold cross-validation. Before implementing the 10-fold configuration, several architectures were tested to ensure satisfactory performance. In each fold, the network was trained for 15 epochs resulting in 10 models (one epoch covers approximately 2.3 million equally distributed positive and negative training samples as well as 0.26 million test samples).

### Implementation of adaptive windowing algorithm

#### Motivation

Figure 2 shows the elementary operation of the proposed windowing algorithm. A window runs along the ECG signal to extract an arbitrary ECG segment consecutively. Then the ECG segment is passed through the trained CNN to calculate the probability *P(B)*, where *B* is the event containing a full heartbeat-related pattern in an ECG segment (length $\le 1.4$ s). If the CNN predict the relevant ECG segment is a Non-valid heartbeat segment, the window is moved forward a step and repeats the same processes until a valid heartbeat ECG segment is met. However, this approach arouses some challenges, as depicted below.

- multiple detections (False Positives) of the same beat can be expected if the step size is too small.
- a larger number of misdetections can be expected if the step size is too long
- none or significantly fewer detections (False Negatives) can be expected if the window size is too long or too short

Therefore, using a fixed window with a fixed step size may cause numerous misdetections, over-detections and malperformance. In addition, after detecting a Valid Heartbeat segment, the boundaries should be defined so that,

- the most appropriate features are preserved
- the main morphology is aligned to the centre (because the CNN detects valid heartbeat segments which are inexplicitly seated around the centre of the segment)
- minimised or zeroed morphological parts integrated into the segmented part from neighbouring heartbeats

All things considered, the facts suggest that the window size, step size and boundary should be meticulously calculated by exploiting the local characteristics and behaviour of the interested region of the signal.

#### Adaptive windowing algorithm, setting boundaries, and beat segmentation

**Figure 7:**
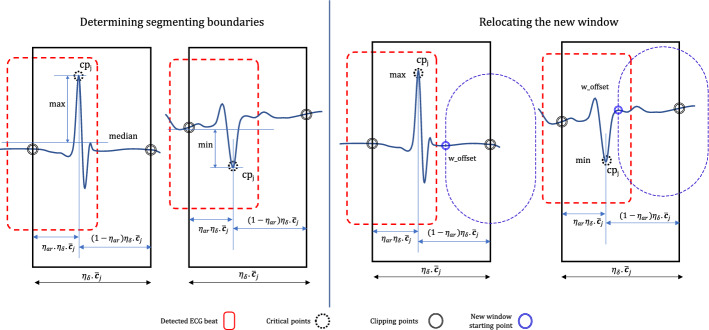
Determination of critical points and window relocation after segmentation.

**Figure 8:**
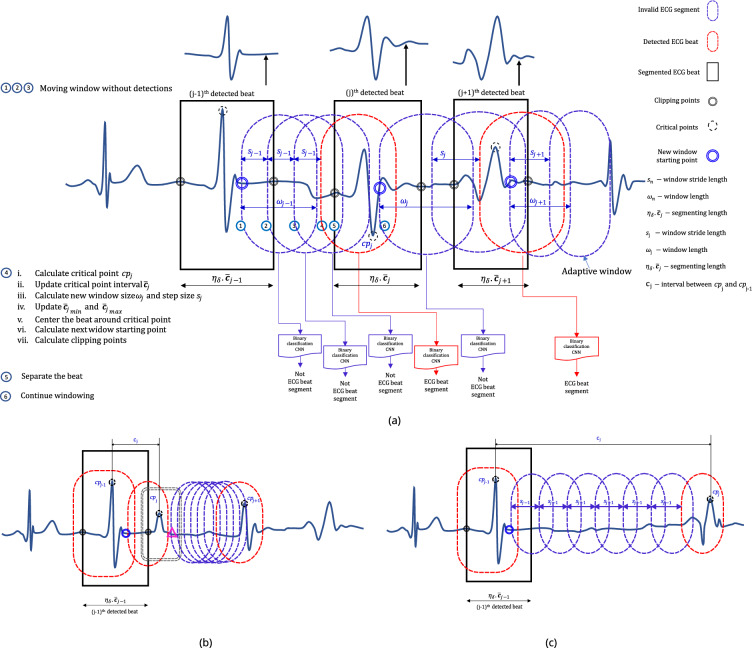
Detailed overview of the adaptive windowing process.

After the $j$th heartbeat detection, the length of the window, and the step size, are denoted as $\omega _j$ and $s_j$ respectively (see Fig. 8a). Initial parameters such as starting window length $\omega _0$, step size $s_0$ etc. are calculated in a separate process (demonstrated later in this article) before executing the segmentation process. Assume that the $({j-1})^{th}$ beat is detected, and then the window is moved forward with $s_{j-1}$ step. Then an ECG segment ${\zeta }_{tmp}$ with a length of $\omega _{j-1}$ is separated, preprocessed, aligned and passed through the CNN to calculate the probability *P*(*B*) (B is the event ${\zeta }_{tmp}$ being a valid heartbeat). If $P(B) > p_b$, where $p_b(=0.9)$ is a predefined confidence level, then it can be safely inferred that most of ${\zeta }_{tmp}$ fully or partially contain a **QRS-like** segment. However, it is obvious that the main morphology may not align with the centre of ${\zeta }_{tmp}$ because the window does not cover the entire event. Therefore, a *cp* is calculated to approximate the point where the main event is centred around. The boundaries can then be calculated based on it. As shown in Fig. 7, the *cp* of the ${\zeta }_{tmp}$ is computed based on the central tendency (here, we chose the median as the central tendency measure) of the segment and the local maximum and the minimum. If the central tendency is closer to the local maximum, the *cp* is considered as the maximum and if it is closer to the local minimum then the *cp* is selected as the local minimum. In the rest of the article, we refer to the *horizontal component* (sample index) of the *cp* as *cp*.

If $P(B) \le p_b$, the window is forwarded without updating window parameters. When $P(B) > p_b$ and $cp_{j}$ is not too close to the previous $cp_{j-1}$, the new window size $\omega _{j}$ and step $s_{j}$ is calculated as a ratio of mean *cp* interval ${\bar{{\textbf {C}}}_{j}}$ resulting $(\eta _w\cdot {\bar{{\textbf {C}}}_{j}}$ and $\eta _s\cdot {\bar{{\textbf {C}}}_{j}})$ respectively, where $\eta _w(=0.9)$ and $\eta _s(=\frac{3\cdot \eta _w}{11})$ are predefined constants. Equation (8) shows how ${\bar{{\textbf {C}}}_{j}}$ is calculated.

Subsequently, left and right boundaries are calculated. Here we propose two cases to calculate the boundaries (method I and II). In method I, the boundaries of the $j{th}$ beat are calculated with reference to the $cp_{j}$, and predefined constants, centre align ratio $\eta _{ar}(=\frac{5}{11})$, safe margin constant $\eta _{\delta } (=\frac{9}{10})$ and $\overline{{{\textbf {C}}}_{j}}$ resulting the segmentation length being $\eta _{\delta }\cdot {\bar{{\textbf {C}}}_{j}}$. Here, the current critical event is aligned so that the *cp* lies in a 5 : 6 ratio within the segmented ECG beat. In method II, the boundaries of $(j-1){th}$ are calculated based on the locations of adjacent (left $cp_{j-2}$ , right $cp_{j}$) and predefined arbitrary constants $\eta _l (e.g., 0.5)$ and $\eta _r (e.g., 0.5)$. Once the segmentation is executed, the new window $\omega _{j}$ starts from a point beyond the current critical point to save iterations and avoid multiple detections. The length of the offset is calculated proportionally to window $\omega _j$ length using a constant $\eta _{of}(=0.1)$.

#### Avoiding false detections caused in exceptional scenarios

If the ECG signal is too noisy or anomalous, multiple detections can be expected in the neighbourhood of current *cp* for Non-heartbeat segments which morphologically appear as **QRS-like** segments (e.g., wider QRS or T wave, deformed T wave etc.). As the window size directly depends on the moving average of the *cp* interval $\overline{{\textbf {C}}}_j$ and updated at each *j*th detection, the adaptive window parameters can be erroneous (may cause the window to be very small) causing many iterations to auto-correct. Figure 8a,b show a double-checking procedure introduced to tackle the trade-off between maintaining the adaptability of the window parameters and avoiding false detection near the main *cp*.

If the newly detected $\tilde{cp}_j$ is too close to the $cp_j$, the same window is run starting from a slightly different point beyond the faulty $\tilde{cp}_j$ with tiny steps until it detects the next *cp*. The faulty $\tilde{cp}_j$ does not take into account when calculating the mean *cp* interval. The faulty $\tilde{cp}_j$ is detected based on adaptively changing thresholds $\overline{cp}_{{min}_{j}}$ and $\overline{cp}_{{max}_{j}}$ where $\overline{cp}_{{min}_{j}} = \eta _{cmin}\cdot \overline{{\textbf {C}}}_{j}, (\eta _{cmin} = 0.45)$ and $\overline{cp}_{{max}_{j}} =\eta _{cmax}\cdot \overline{{\textbf {C}}}_{j}, (\eta _{cmax} = 1.45)$. If a $\tilde{cp}_j$ is detected within 45% of $\overline{{\textbf {C}}}_{j-1}$ or beyond 145% of $\overline{{\textbf {C}}}_{j-1}$, it infers that the $\tilde{cp}_j$ is too close or too far to the ${cp}_{j-1}$. Therefore, all $\tilde{cp}_j$ that do not satisfy these constraints are omitted when calculating the new mean *cp* interval $\overline{{\textbf {C}}}_j$. On the other hand, if the current *cp* is too far away from the last detected ${cp}_{j-1}$, it also causes the window to be too large resulting in no detections or faulty detection. Therefore, an adaptively changing threshold is calculated $\overline{cp}_{{max}_{j}} = \eta _{cmax}\cdot {\bar{{\textbf {C}}}_{j}} (\eta _{cmax} = 1.4$ means that if there is a *cp* within 145% of mean *cp* interval $\overline{{\textbf {C}}}_{j-1}$, then $\tilde{cp}_j$ is omitted when calculating the new mean *cp* interval ${\bar{{\textbf {C}}}_{j}})$. As a result, abruptly emerging false ***QRS-Like*** events within reach of the main *cp* or too far away from the *cp* have no major influence on miscalculating the window parameters. However, the *cp*s found too far are segmented using current window parameters.

**Figure 9:**
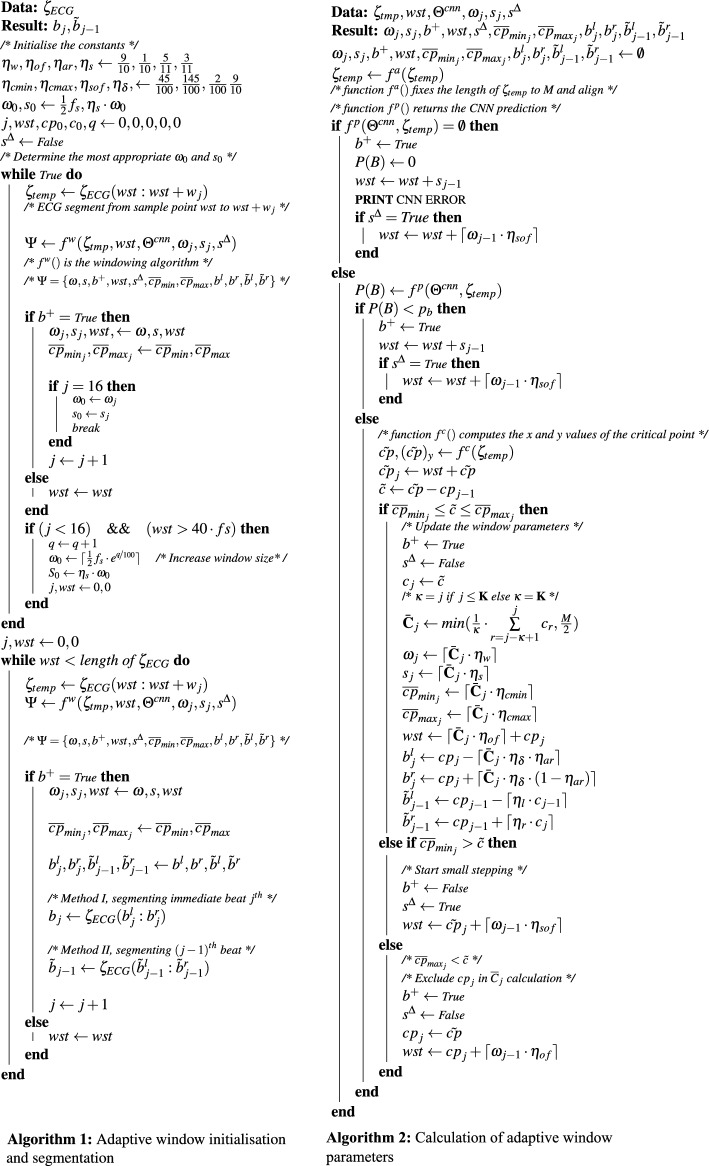
Pseudo-codes for the proposed algorithms.

Equation (5) shows how the window parameters are updated in occurrences of valid beat detection and how the boundaries are calculated for method I. Equation (6) shows how the boundaries are calculated for ${(j-1)}{th}$ beat after the detection of *j* *th* beat (method II). Similarly Eq. (7) shows how the parameters get updated when a valid beat is not detected. Algorithm 1 (see Fig. 9) shows the pseudo-code for computing the initial window parameters before starting the segmentation, as the window is not yet adapted. Here we run the algorithm for the first 16 beats without performing segmentation. The initial window size $\omega _0$ is set as $\frac{1}{2}\cdot f_s$ (0.5 s) where $f_s$ is the sampling rate. Then the window size is updated in each detection. If the window is not correctly adapted after the 16*th* beat, the initial window size $\omega _0$ is increased by multiples of $e^{0.01}$. Algorithm 2 (see Fig. 9) shows the pseudo-code of how the window parameters are updated during each iteration.

5 $$\begin{aligned}{} & {} \begin{bmatrix} \omega _j \\ s_j \\ wst\\ \overline{cp}_{{min}_{j}} \\ \overline{cp}_{{max}_{j}} \\ {b}_{j}^l \\ {b}_{j}^r \end{bmatrix} = {\bar{{\textbf {C}}}_{j}} \begin{bmatrix} \eta _w \\ \eta _s \\ \eta _{of} \\ \eta _{cmin}\\ \eta _{cmax}\\ -\eta _\delta \cdot \eta _{ar} \\ \eta _\delta \cdot (1-\eta _{ar})\\ \end{bmatrix} + \begin{bmatrix} 0 \\ 0 \\ cp_j \\ 0\\ 0\\ cp_j \\ cp_j \end{bmatrix} \end{aligned}$$

6 $$\begin{aligned}{} & {} \quad \begin{bmatrix} {\tilde{b}}_{j-1}^l \\ {\tilde{b}}_{j-1}^r \end{bmatrix} = \begin{bmatrix} {cp}_{j-1} \\ {cp}_{j-1} \end{bmatrix} + \begin{bmatrix} -{c}_{j-1} \\ {c}_{j} \end{bmatrix} \bigodot \begin{bmatrix} \eta _{l} \\ \eta _{r} \end{bmatrix} \end{aligned}$$

7 $$\begin{aligned}{} & {} \quad \begin{bmatrix} \omega _j \\ s_j \\ wst \\ \\ \overline{cp}_{{min}_{j}} \\ \overline{cp}_{{max}_{j}} \\ {b}_{j}^l \\ {b}_{j}^r \\ \tilde{b}_{j-1}^l \\ \tilde{b}_{j-1}^r \end{bmatrix} = \begin{bmatrix} \emptyset \\ \emptyset \\ {\left\{ \begin{array}{ll} wst + s_{j-1} &{}\text { if } P(B)< p_b\\ \tilde{cp} + \delta &{}\text {if } P(B)\ge p_b\\ \end{array}\right. } \\ \emptyset \\ \emptyset \\ \emptyset \\ \emptyset \\ \emptyset \\ \emptyset \end{bmatrix} \end{aligned}$$

8 $$\begin{aligned}{} & {} \quad {\bar{{\textbf {C}}}_{j}} = \frac{1}{\kappa }\sum _{r=j-\kappa +1}^{j} {c_r};\quad j \ge 0, j \ge \kappa , \kappa \ne 0 \end{aligned}$$

where, $c_r$ is the interval between $c_r$ and $c_{r-1}$, $\kappa = K (=16)$, $j=k$ in initialising phase (when $\kappa \le K$)

For quantitative analysis, the proposed method is evaluated using the MIT-BIH arrhythmia database, the European ST-T database, and Fantasia database. The CNN model was trained, tested and tuned since the MIT-BIH arrhythmia database contains numerous anomalous QRS complexes, irregular rhythmic patterns, significant baseline drifts, and rapid changes. The performance is evaluated in several steps as follows.

1. Performance evaluation for trained CNN based on 10-fold configuration for MIT-BIH arrhythmia database
2. Performance evaluation for adaptive windowing algorithm on MIT-BIH arrhythmia database
  - Evaluation of the accuracy of locating critical points
  - Conformity of the boundaries of each beat based on manual inspection
3. Performance analysis of detecting critical points on the European ST-T database and Fantasia database (unseen data for the CNN)

It is practically impossible to formulate a coherent criterion to assess the accuracy of the boundaries. So, the segmented ECG beats were manually inspected to ensure that the boundaries were satisfactorily defined in accordance to a checklist (qualitative analysis) as follows. *qualifying criterion*

1. the critical point (main ECG event) is sufficiently aligned to the centre of the segmented ECG beats
2. the left and right margins are lied on the isoelectric line without overlapping the nearby ECG beats when applicable
3. slightly overlapped or trivially truncated versions of ECG beats were passed as correct when,
  - the margin of a beat is not straightforward or ambiguous
  - the isoelectric line is not presented clearly due to the irregular nature of abnormal patterns, missing QRS patterns or possible intermingling between adjacent cardiac cycles

*disqualifying criterion*

1. when multiple critical points are observed in a segmented portion
2. when there is substantial overlap, and the boundaries are unambiguous
3. When a clear *QRS-Like* morphology is not observed in a segmented portion
4. when *QRS-Like* morphology is substantially aligned towards left or right corners

The European ST-T database and Fantasia database were used as a validation dataset, to verify the algorithm’s ability to locate critical points accurately. However, a subjective inspection was not performed as the dataset is too large.

## Results

### Performance of the CNN model

The average accuracy for the whole dataset is determined based on the average accuracy of the test dataset of each fold. The average accuracy of the CNN is determined to be 99.11%. The fold wise accuracies showed almost consistent test accuracy for each fold, proving that the CNN is robust. According to Table 2, CNN has accurately classified 1,299,606 valid ECG beats and 1,307,380 Non-valid ECG beats. Here, we evaluated all alternative ECG segments when calculating the matrices, as they are not subject to any alterations other than shifting and trimming. However, there is no overlap between the training and test datasets as the folds are configured record-wise. The sensitivity, accuracy, specificity , and the F1 score for the proposed model were calculated as 99.03%, 99.08%, 99.12%, and 99.08%, respectively.

**Figure 10:**
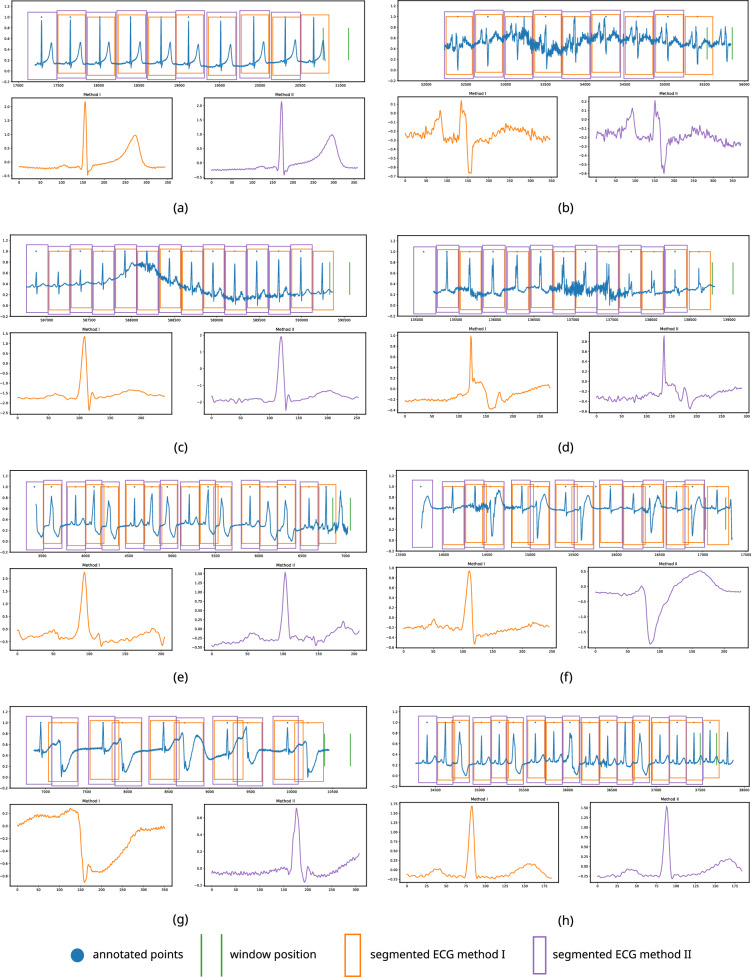
Segmentation of heartbeats from (**a**) a good quality signal comprised with normal heartbeats (record 113) (**b**) a noisy signal comprised of right bundle branch block beats (record 108) (**c**) a signal which has a baseline wander comprised with normal heartbeats (record 116) (**d**) a noisy signal comprised with normal heartbeats (record 104) (**e**) a signal comprised with premature ventricular contraction and fusion of the ventricular and normal beat (record 208) (**f**) a signal comprised with premature ventricular contraction (record 200) (**g**) a signal comprised with right bundle branch block beats and premature ventricular contraction (record 207) (**h**) a signal comprised with abnormal heartbeats (record 208).

**Figure 11:**
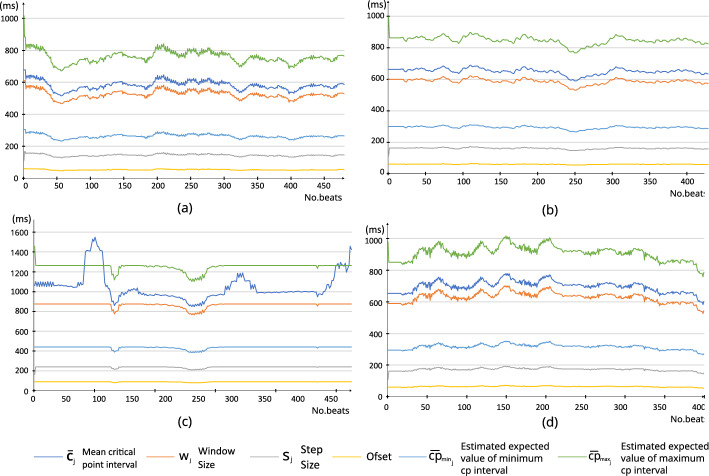
Adapting window parameters in each detection (**a**) record 208 (**b**) record 212 (**c**) record 207 (**d**) record 200.

**Table 2:** Performance matrix for the proposed CNN.

		Predicted labels	
		No. of invalid beats	No. of valid beats
True Labels	No. of invalid beats	**1299606**	11474
	No. of valid beats	12850	**1307380**
	Sensitivity (Se, %)	99.03	
	Precision (P+, %)	99.13	
	Specificity (Se, %)	99.12	
	F1 Score (F1, %)	99.08	
	Accuracy (%)	99.08	

Numbers of correct predictions are given in bold.

### Performance of the ECG segmentation

In evaluating the segmentation, some parts of record 207 were discarded as we decided that the critical points were too ambiguous. Similarly, seven records (e0112, e0129, e0133, e0304, e0305, e0415, e0604) were not considered, as some annotations seem inconsistent. Whole segmentation algorithm was also evaluated based on the criteria explained in this article. Table 3 shows the number of True Positives and False Positives, the precision and the accuracy for each record. As this algorithm focuses on segmenting the ECG beat, we used the term critical point, to separate the main ECG event accurately. Therefore, some detected critical points, match the given annotated locations within a range. However, the majority of annotated locations exactly match the detected location. Even though this study does not focus on finding precise R-peaks, we decided to compare the peak locations with our critical points as tabulated in Table 3 for the sake of relating our research to similar studies. In Table 3, we illustrated the performance of locating the critical points and the segmentation performance.

**Table 3:** Performance of the critical point detection and ECG segmentation algorithm on MIT-BIH arrhythmia database.

Fold	Record	Annotated *CP*s	Detected *CP*s	Locating critical points												Segmentation			
				TP(±50ms)	FP($\pm 50ms$)	ACC.(%)	$+P(\%)$	TP($\pm 100ms$)	FP($\pm 100ms$)	ACC.(%)	$+P(\%)$	TP$(\pm 150ms)$0	FP$(\pm 150ms)$	ACC.%	$+P(\%)$	Improper boundaries	Proper boundaries	ACC.(%)	$+P(\%)$
1	210	2650	2645	2633	12	99.4	99.5	2633	12	99.4	99.5	2633	12	99.4	99.5	12	2633	99.4	99.5
	221	2427	2428	2412	16	99.4	99.3	2412	16	99.4	99.3	2412	16	99.4	99.3	7	2421	99.8	99.7
	123	1518	1505	1505	0	99.1	100.0	1505	0	99.1	100.0	1505	0	99.1	100.0	2	1503	99.0	99.9
	213	3251	3244	3212	32	98.8	99.0	3242	2	99.7	99.9	3242	2	99.7	99.9	3	3241	99.7	99.9
2	214	2260	2261	2150	111	95.1	95.1	2247	14	99.4	99.4	2247	14	99.4	99.4	17	2244	99.3	99.2
	105	2567	2530	2460	70	95.8	97.2	2473	57	96.3	97.7	2480	50	96.6	98.0	37	2493	97.1	98.5
	102	2187	2187	2121	66	97.0	97.0	2178	9	99.6	99.6	2178	9	99.6	99.6	0	2187	100.0	100.0
	116	2412	2393	2388	5	99.0	99.8	2388	5	99.0	99.8	2390	3	99.1	99.9	1	2392	99.2	100.0
	111	2124	2129	2085	44	98.2	97.9	2087	42	98.3	98.0	2087	42	98.3	98.0	5	2124	100.0	99.8
3	208	2953	2932	2918	14	98.8	99.5	2921	11	98.9	99.6	2922	10	99.0	99.7	4	2928	99.2	99.9
	231	1571	1643	1554	89	98.9	94.6	1554	89	98.9	94.6	1555	88	99.0	94.6	8	1635	104.1	99.5
	212	2748	2741	2741	0	99.7	100.0	2741	0	99.7	100.0	2741	0	99.7	100.0	0	2741	99.7	100.0
	106	2027	2015	1964	51	96.9	97.5	1988	27	98.1	98.7	1990	25	98.2	98.8	35	1980	97.7	98.3
	219	2154	2137	2135	2	99.1	99.9	2135	2	99.1	99.9	2135	2	99.1	99.9	1	2136	99.2	100.0
4	223	2605	2602	2597	5	99.7	99.8	2599	3	99.8	99.9	2599	3	99.8	99.9	2	2600	99.8	99.9
	205	2656	2641	2636	5	99.2	99.8	2636	5	99.2	99.8	2636	5	99.2	99.8	3	2638	99.3	99.9
	117	1535	1532	1527	5	99.5	99.7	1527	5	99.5	99.7	1527	5	99.5	99.7	1	1531	99.7	99.9
	222	2483	2456	2437	19	98.1	99.2	2438	18	98.2	99.3	2438	18	98.2	99.3	15	2441	98.3	99.4
	233	3079	3065	3047	18	99.0	99.4	3064	1	99.5	100.0	3065	0	99.5	100.0	1	3064	99.5	100.0
5	114	1879	1852	1819	33	96.8	98.2	1819	33	96.8	98.2	1819	33	96.8	98.2	6	1846	98.2	99.7
	232	1780	1790	1767	23	99.3	98.7	1767	23	99.3	98.7	1767	23	99.3	98.7	16	1774	99.7	99.1
	107	2137	2135	1735	400	81.2	81.3	1780	355	83.3	83.4	2134	1	99.9	100.0	3	2132	99.8	99.9
	104	2211	2207	2115	92	95.7	95.8	2188	19	99.0	99.1	2193	14	99.2	99.4	5	2202	99.6	99.8
	234	2753	2743	2743	0	99.6	100.0	2743	0	99.6	100.0	2743	0	99.6	100.0	0	2743	99.6	100.0
6	207	1872	1822	1783	39	95.2	97.9	1806	16	96.5	99.1	1808	14	96.6	99.2	59	1763	94.2	96.8
	122	2476	2469	2469	0	99.7	100.0	2469	0	99.7	100.0	2469	0	99.7	100.0	0	2469	99.7	100.0
	201	1963	1934	1753	181	89.3	90.6	1760	174	89.7	91.0	1860	74	94.8	96.2	8	1926	98.1	99.6
	217	2208	2188	1115	1073	50.5	51.0	2181	7	98.8	99.7	2181	7	98.8	99.7	4	2184	98.9	99.8
	209	3005	3006	3002	4	99.9	99.9	3002	4	99.9	99.9	3002	4	99.9	99.9	1	3005	100.0	100.0
7	203	2976	2886	2761	125	92.8	95.7	2783	103	93.5	96.4	2883	3	96.9	99.9	17	2869	96.4	99.4
	202	2136	2079	2056	23	96.3	98.9	2056	23	96.3	98.9	2056	23	96.3	98.9	22	2057	96.3	98.9
	228	2053	2034	1982	52	96.5	97.4	1995	39	97.2	98.1	1995	39	97.2	98.1	15	2019	98.3	99.3
	119	1987	1980	1980	0	99.6	100.0	1980	0	99.6	100.0	1980	0	99.6	100.0	13	1967	99.0	99.3
	230	2256	2253	2253	0	99.9	100.0	2253	0	99.9	100.0	2253	0	99.9	100.0	0	2253	99.9	100.0
8	115	1953	1945	1945	0	99.6	100.0	1945	0	99.6	100.0	1945	0	99.6	100.0	0	1945	99.6	100.0
	100	2273	2260	2260	0	99.4	100.0	2260	0	99.4	100.0	2260	0	99.4	100.0	0	2260	99.4	100.0
	215	3363	3360	3357	3	99.8	99.9	3357	3	99.8	99.9	3357	3	99.8	99.9	0	3360	99.9	100.0
	124	1619	1678	1608	70	99.3	95.8	1617	61	99.9	96.4	1617	61	99.9	96.4	21	1657	102.3	98.7
	101	1863	1882	1857	25	99.7	98.7	1857	25	99.7	98.7	1857	25	99.7	98.7	4	1878	100.8	99.8
9	103	2084	2073	2073	0	99.5	100.0	2073	0	99.5	100.0	2073	0	99.5	100.0	1	2072	99.4	100.0
	118	2278	2272	2229	43	97.8	98.1	2271	1	99.7	100.0	2271	1	99.7	100.0	1	2271	99.7	100.0
	200	2601	2592	2567	25	98.7	99.0	2584	8	99.3	99.7	2584	8	99.3	99.7	1	2591	99.6	100.0
	220	2048	2048	2044	4	99.8	99.8	2044	4	99.8	99.8	2044	4	99.8	99.8	3	2045	99.9	99.9
	113	1795	1783	1783	0	99.3	100.0	1783	0	99.3	100.0	1783	0	99.3	100.0	1	1782	99.3	99.9
10	108	1763	1739	1644	95	93.3	94.5	1687	52	95.7	97.0	1687	52	95.7	97.0	19	1720	97.6	98.9
	121	1863	1858	1845	13	99.0	99.3	1846	12	99.1	99.4	1847	11	99.1	99.4	1	1857	99.7	99.9
	109	2532	2524	2509	15	99.1	99.4	2524	0	99.7	100.0	2524	0	99.7	100.0	1	2523	99.6	100.0
	112	2539	2536	2536	0	99.9	100.0	2536	0	99.9	100.0	2536	0	99.9	100.0	5	2531	99.7	99.8
Total		109473	109014	106112	2902			107734	1280			108310	704			381	108633		
Average						96.93	97.34			98.41	98.83			98.94	99.35			99.25	99.62

**Table 4:** Performance of the critical point detection algorithm on MIT-European ST-T database ($\pm 25$ ms margin basis).

Record	Annotated *CP*s	Detected *CP*s	TP	FP	$P^+$	ACC.%	Record	Annotated *CP*s	Detected *CP*s	TP	FP	$P^+$	ACC.%
e0103	7311	7275	7266	9	99.9	99.4	e0212	10835	10826	10807	19	99.8	99.7
e0104	7747	7694	7685	9	99.9	99.2	e0213	11079	11086	11057	29	99.7	99.8
e0105	6683	6611	6148	463	93.0	92.0	e0302	10355	10301	10276	25	99.8	99.2
e0106	7197	7056	6983	73	99.0	97.0	e0303	8880	8873	8865	8	99.9	99.8
e0107	7077	7000	6983	17	99.8	98.7	e0306	7927	7858	7776	82	99.0	98.1
e0108	6687	6589	6192	397	94.0	92.6	e0403	9321	9296	9293	3	100.0	99.7
e0110	7000	6966	6807	159	97.7	97.2	e0404	6987	6940	6937	3	100.0	99.3
e0111	7565	7433	7323	110	98.5	96.8	e0405	11163	11092	11090	2	100.0	99.3
e0113	9057	8947	8930	17	99.8	98.6	e0406	8963	8762	8734	28	99.7	97.4
e0114	5629	5569	5569	5540	50.1	98.9	e0408	9043	9036	9032	4	100.0	99.9
e0115	11319	13260	11311	1949	85.3	99.9	e0409	12889	12887	12880	7	99.9	99.9
e0116	4517	4502	4447	55	98.8	98.5	e0410	7542	7523	7520	3	100.0	99.7
e0118	7121	7080	7080	0	100.0	99.4	e0411	9955	9927	9897	30	99.7	99.4
e0119	7764	7727	7656	71	99.1	98.6	e0413	8164	8146	8056	90	98.9	98.7
e0121	10658	10623	10618	5	100.0	99.6	e0417	9262	9252	9249	3	100.0	99.9
e0122	11387	11363	11362	1	100.0	99.8	e0418	11727	13591	11705	1886	86.1	99.8
e0123	9190	9174	9174	0	100.0	99.8	e0501	7776	7751	7737	14	99.8	99.5
e0124	9249	9213	9213	0	100.0	99.6	e0509	8091	8089	8085	4	100.0	99.9
e0125	9093	9069	9055	14	99.8	99.6	e0515	10748	12146	10651	1495	87.7	99.1
e0126	8300	16121	8266	7855	51.3	99.6	e0601	8789	8767	8762	5	99.9	99.7
e0127	9427	9391	9389	2	100.0	99.6	e0602	11152	11112	11045	67	99.4	99.0
e0136	7083	6929	6868	61	99.1	97.0	e0603	7990	7847	7472	375	95.2	93.5
e0139	10646	10386	10233	153	98.5	96.1	e0605	11389	11337	11311	26	99.8	99.3
e0147	6398	6362	6356	6	99.9	99.3	e0606	9650	9624	9609	15	99.8	99.6
e0148	6708	6614	6547	67	99.0	97.6	e0607	10284	10270	10256	14	99.9	99.7
e0151	7574	7548	7545	3	100.0	99.6	e0609	9333	9321	9319	2	100.0	99.8
e0154	6788	6288	5880	408	93.5	86.6	e0610	8019	7999	7998	1	100.0	99.7
e0155	8137	7829	7217	612	92.2	88.7	e0611	5812	5817	5812	5	99.9	100.0
e0159	9199	7862	7676	186	97.6	83.4	e0612	6902	6879	6876	3	100.0	99.6
e0161	8872	8857	8856	1	100.0	99.8	e0613	7803	7725	7724	1	100.0	99.0
e0162	10634	10613	10591	22	99.8	99.6	e0614	11143	11107	10981	126	98.9	98.5
e0163	7622	7594	7547	47	99.4	99.0	e0615	7202	7193	7191	2	100.0	99.8
e0166	6434	6399	6398	1	100.0	99.4	e0704	9744	10298	9356	942	90.9	96.0
e0170	8833	8823	8819	4	100.0	99.8	e0801	9403	9392	9385	7	99.9	99.8
e0202	9892	9857	9831	26	99.7	99.4	e0808	11108	11067	10949	118	98.9	98.6
e0203	10177	10166	10162	4	100.0	99.9	e0817	7563	7170	6823	347	95.2	90.2
e0204	11484	11464	11418	46	99.6	99.4	e0818	10141	10130	10129	1	100.0	99.9
e0205	11827	11101	11060	41	99.6	93.5	e1301	8761	8727	8594	133	98.5	98.1
e0206	10949	10903	10872	31	99.7	99.3	e1302	8374	8337	8333	4	100.0	99.5
e0207	7218	7188	7169	19	99.7	99.3	e1304	7888	7850	7697	153	98.1	97.6
e0208	8704	8696	8690	6	99.9	99.8		Total				Average	
e0210	8745	8727	8717	10	99.9	99.7		738054	745143	726095	24588	97.4	98.3
e0211	14995	14923	14917	6	100	99.5							

**Figure 12:**
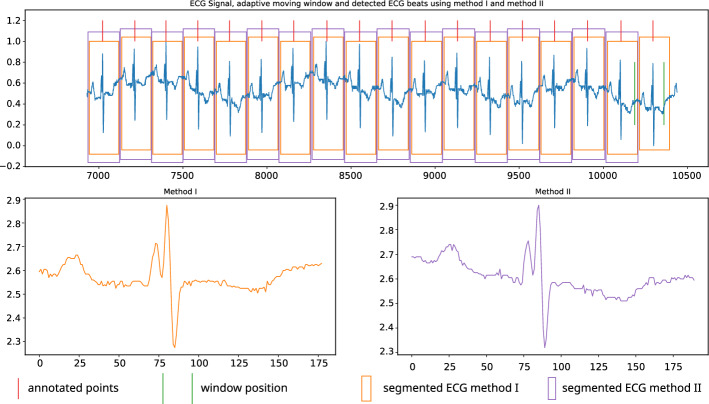
Segmentation examples (European ST-T database record e0139).

The average agreement of detecting a critical point and an annotated location is 96.93, 98.41 and 98.94% within $\pm 25, \pm 50$, and $\pm 75$ (ms) margins, respectively. The study in^16^ also used this kind of margin criteria to evaluate the performance. 108,633 beats out of 109,473 have been correctly identified and appropriately segmented in this study. The average of correctly identified and segmented beats is 99.25%, and the precision for correctly segmented beats is 99.62%. Figure 10 shows an assortment of examples which proves the robustness of the proposed algorithm against various scenarios of ECG signals. In each sub-figure, in Fig. 10, the top figure shows the detected ECG beat and the boundaries calculated based on method I and method II, and the bottom two figures show the segmented ECG beats (last two beats). Two green vertical lines show the size of the next window and its position. For demonstration purposes, we indicated the annotated points with a blue dot provided by the original database. Figure 12 shows an instance of heartbeat segmentation from the European ST-T database. Here, red vertical lines indicate the given annotation location, and green vertical lines indicate the window size and position. The proposed algorithm perfectly detects and segments the ECG beats even when the signals are heavily affected by practical issues such as noise, baseline wander, abnormally larger or smaller S-T waves, morphological disparities, abnormally larger or smaller RR intervals, abnormally suppressed QRS patterns or irregular wave patterns which are illustrated in Figs. 10 and 12. Figure 11 shows how the windowing parameters such as window size, step size, windowing start offset, and expected maximum/ minimum *cp* interval, adaptively follow the fluctuations of mean critical point interval in different ratios.

**Table 5:** Results comparison of the proposed approach with other databases, and *P. Silva et al.* work in^33^.

Database	QRS detection performance					
	Pan-Tompkins		P.silva et el.*		Proposed Method**	
	P+	F1-score	P+	F1-score	P+	Acc.%
CY-BHI	90.28	0.93	96.77	0.96	-	–
MIT-BIH	97.84	0.97	100	0.96	**99.13**	**99.08**
ST-T	–	–	–	–	**97.74**	**98.30**
Fantasia	–	–	–	–	**99.37**	**99.37**

*The performance is calculated only for healthy subjects.

**The performance is calculated for all subjects including healthy and unhealthy

## Discussion

In this work, we proposed two ECG beat segmentation methods using a CNN model and an adaptive windowing algorithm that can serve as a preprocessing tool in beat-wise ECG analysing algorithms. This research was performed to improve the precision of ECG segmentation so that abnormal ECG beats also can be segmented. Specifically, we used a CNN model to detect critical points where the main ECG morphology is formed around to recognise an occurrence of a heartbeat cycle, unlike other methods, which employ signal quality, filters, or other signal processing techniques such as peak detection etc. Therefore a complete or incomplete heart cycle, including abnormal patterns such as arrhythmic events, could be identified more accurately. The performance matrix in Table 2 shows that the specificity, sensitivity, precision and F1-Score are close to 100%, meaning that the CNN model is very confident in classifying heartbeat and Non-heartbeat segments. As many similar studies focus on detecting accurate R peaks/ QRS detection and the proposed method focuses on adaptive segmentation via detecting critical points, this work differs from those in some aspects. So we compare our work with the study^33^ in Table 5 as both studies used the same database, and some techniques are comparable to each other.

In contrast to the CNN model proposed in^33^, our model achieved an F1-score of 99.8% whilst their model is 96% for the MIT-BIH dataset. The precision shows a slightly lower value of 99.13% to their 100%. However, it should be noted that our model can distinguish both normal and abnormal heartbeat compared to the study in^33^, where the model is trained only with healthy individuals (only 23 records). Furthermore, in^33^ the training data is prepared using fixed lengths from the annotated points, unlike ours where all the lengths are calculated locally with reference to the adjacent critical points. This makes our CNN to be more sensitive to wider or narrower variations of QRS morphology. Additionally, the proposed model demonstrates better performance in terms of sensitivity, precision, and F1-Score in comparison to the Pan-Tompkins algorithm in locating critical points.

In calculating the boundaries of the heartbeat, we used an instantaneous critical point interval which can be closely related to the RR interval. Results presented in Tables 3 and 4 show that our idea of using an adaptive window calculated based on mean *cp* interval to identify **QRS-like** patterns and determine boundaries is a success.

Figure 10b–d,f demonstrate that the proposed algorithm can successfully detect and segment regular and irregular heartbeats even if the signal comprises abrupt changes, baseline wander, or a considerable level of noise, resulting in a high number of true positive and true negative detection level whilst having very low false negative and false positive. Figure 10a,e,g,h also show the observation in detecting and segmenting atypical heartbeats such as premature ventricular contraction etc. The algorithm showed appealing average accuracy of 98.3% and a precision of 97.4% for the unseen dataset as illustrated in Fig. 12 and Table 4.

The reported results for both datasets suggest a high possibility of using this algorithm in ECG analysing as a preprocessing tool, given the notion that correct segmentation is critical for medical equipment and the arrhythmia classification algorithms.

Even though some studies^35,36^ performed for R peak detection and detailed ECG delineation can not be directly related to our work, we review some potentials specific to this study for comparison and discussion. High pass or low pass filtering techniques were not exploited in our work to denoise the signal in contrast to the work in^37^, which was performed to detect T-Wave. Unlike in^37^, the proposed algorithm can be directly applied to the raw signal. On the other hand, our algorithm adapts its parameters depending on the *cp* interval in each detection, allowing to use of this algorithm for a wide variety of ECG waves which shows different characteristics.

Getting a high positive prediction rate is important to avoid false detections in many ECG applications. As a result of using adaptive window size and step size, we could use the sliding window more efficiently to reduce the number of iterations per detection (it can be fewer steps, 1–3 depending on the nature of the ECG). In this study, we did not use any hard thresholds allowing the algorithm to be adapted to the interested region of the signal. The window parameters and other local points are always recalculated and updated in each detection allowing the algorithm to detect the next heartbeat smoothly. As we tested the algorithm with two datasets, we found that the algorithm shows outstanding performance for unseen data, proving that this algorithm can be used robustly in detecting and segmenting ECG signals. In addition, the segmentation performance is monitored manually, beat by beat, to ensure that the boundaries are reasonable. It is important to mention that the high sensitivity and positive prediction rate reported in the CNN model proposed in this study have a balanced trade-off that supports the notion that this algorithm can be used reliably and accurately as an ECG segmentation tool.

## Limitations and future works

The proposed approach intends to distinguish ECG beats from an ECG signal, including normal and pathological beats. However, the CNN model is trained based on one dataset; therefore, some pathological patterns might be new to the model, which may lead it to perform differently than intended. Possible failures of the windowing algorithm can be expected when extraordinarily high or low RR intervals are met as the CNNs maximum input is limited to 1.4 s. However, this problem rarely arises as the RR interval usually is lower than 1.4 s. There is a space to fine-tune the constants based on practical observations, domain-based knowledge and specific case studies. Safety mechanisms such as time outs, and checking signal noise levels also can be employed in serious practical cases to ensure the adaptive parameters always lie within the realistic values.

In the future, we plan to train the network with more data collected locally and use other public datasets to increase performance and robustness. Further, the windowing algorithm can be modified in multiple ways to overcome the limitations of this work mentioned in the limitation section. For example, the same CNN model can be employed repeatedly to confirm that the boundaries are reasonable. If the validation fails, a boundary re-adjusting procedure can be implemented based on the prediction score. Multiple segmentation of the same beat is also an option in heavily complicated cases such as incomplete arrhythmic episodes. In future, we aim to extend this algorithm as a vote-based detection system with multiple classification methods to be used in various ECG analysing applications such as^38^ patented by the same authors.

## Data Availability

The data used to support the findings of this study are available freely at https://physionet.org/content/mitdb/1.0.0/ , https://physionet.org/content/edb/1.0.0/, and https://physionet.org/content/fantasia/1.0.0/.
